# Supplementary material for: SAR296968, a Novel Selective Na+/Ca2+ Exchanger Inhibitor, Improves Ca2+ Handling and Contractile Function in Human Atrial Cardiomyocytes
Source: Biomedicines. 2022 Aug 9;10(8):1932. doi: 10.3390/biomedicines10081932 (PMC9406204; doi:10.3390/biomedicines10081932)
Supplement: Supplementary file 1 [file biomedicines-10-01932-s001.zip › Supplemental Material.pdf]

Supplemental Material

SAR296968, a novel selective Na^+/Ca^{2+} exchanger inhibitor, improves Ca^{2+} handling and contractile function in human atrial cardiomyocytes

Philipp Hegner, M.D. [1], Marzena Drzymalski, M.D.[1], Alexander Biedermann[1], Bernadette Memmel[1], Melanie Durczok[1], Michael Wester, M.D.[1], Bernhard Floerchinger, M.D.[2], Zdenek Provaznik, M.D.[2], Christof Schmid, M.D.[2], York Zausig, M.D.[3], Lars S. Maier, M.D.[1], Stefan Wagner, M.D.[1]

[1]Dept of Internal Medicine II, University Medical Center Regensburg, Germany, [2]Dept of Cardiothoracic Surgery, University Medical Center Regensburg, Germany, [3]Department of Anesthesiology, University Medical Center Regensburg, Germany

Correspondence: Prof. Dr. Stefan Wagner
University Medical Center Regensburg
Dept. of Internal Medicine II
Franz-Josef-Strauß-Allee 11
Germany
Tel: +49-941-944-7206
Fax: +49-941-944-7213

Category: Original article

MATERIALS AND METHODS

Human myocyte isolation:

All procedures were performed according to the Declaration of Helsinki and were approved by the local ethics committee. All patients gave written informed consent prior to study inclusion. Biopsies of human right atrial appendages of patients undergoing aorto-coronary bypass grafting were used. Chunk isolation was performed with sliced tissue incubated at 37°C in a spinner flask filled with Tyrode solution (in mmol/L 100 NaCl, 10 KCl, 5 $MgCl_2$, 0.02 $CaCl_2$, 1.2 KH_2PO_4, 50 Taurin, 5 MOPS, 10 BDM, 20 glucose, pH 7.2) with collagenase 1 and protease (type XXIV, 0.04%, Sigma). After 45 min, the supernatant was discarded and the remaining tissue poured into the flask. A second digestion step with Tyrode solution with collagenase 1 (without protease) was performed for 20-35 min (at 37°C). The cells were disaggregated using a Pasteur pipette with a wide tip opening and subsequently centrifuged with 95 g for 10 min at room temperature. The pellet was resuspended in storage solution containing in mmol/L: 30 KCl, 10 KH_2PO_4, 1 $MgCl_2$, 10 HEPES, 11 glucose, 20 taurine, 70 glutamic acid, 20 BDM, 2% BCS, pH 7.4, at room temperature. Cells were then stored in solution that stops enzyme activity containing JMEM and BCS (10%) at room temperature. Only elongated cells with cross striations and without granulation were selected for experiments.

Measurement of Ca^{2+} sparks

Myocytes on laminin-coated recording chambers were loaded with 10 µmol/L fluo-4 acetoxymethylester in the presence of 0.02% (w/v) pluronic acid (Molecular Probes; 30 min incubation), mounted on stage of a laser-scanning confocal microscope (LSM5, Zeiss) and superfused with normal Tyrode (NT) solution containing (in mM) 140 NaCl, 4 KCl, 5 HEPES, 1 $MgCl_2$, 10 glucose, 2 $CaCl_2$ (pH 7.4, room temperature with NaOH). For each patient, separate recording chambers were exposed to either vehicle, 300 nM SAR296968 or 3 µM SAR296968 during the 30 min incubation period and throughout the data acquisition on stage of the microscope. Due to the challenging technique of human atrial cardiomyocyte isolation, limited availability of biomaterial and often small probe size, it was not always possible to measure all experimental groups for each patient. Fluo-4 was excited at 488 nm and emission was collected through a 505 nm long-pass filter. Fluorescence images were recorded in line-scan mode with 512 pixels

per 35.5 µm wide scanline (pixel size 0.07 µm, 1319 lines per second, 10000 lines per scan). Following cessation of 1 Hz stimulation (at 20 V), Ca^{2+}-sparks were measured at resting conditions. [Ca]i was calibrated using the pseudo-ratio equation $[Ca]_i=K_d(F/F_0)/(K_d/[Ca]_{i\text{-}rest}+1–F/F_0)$ with K_d=1,100 nmol/L (estimating $[Ca]_{i\text{-}rest}$ at 100nmol/L) [1]. The diastolic Ca leak was calculated by estimating Ca fluxes upon Ca sparks (J in mol/s) using the equation J = B x Δ[Ca] x V x T-1 with B being the buffering power of the cell (in this case B = 150), Δ[Ca] being the concentration change during a spark, V being the volume occupied by a spark and T being the time taken for rise of Ca during a spark [1]. The Ca flux of each spark was then integrated, summarized for all sparks of a line scan and normalized to scanned cytosolic volume (excluding mitochondria), which give a dimension of µmol/L cytosol/s. Ca^{2+} sparks were analyzed using the Sparkmaster plugin for Image J.
For some experiments, caffeine (10 mmol/L) was applied to induce rapid SR Ca^{2+} release and resulting transients were analyzed.

Patch-clamp experiments
Myocytes were mounted on the stage of a microscope (Zeiss Axio Observer). Ruptured-patch whole-cell current-clamp was used to measure cardiac membrane potential. Microelectrodes (~5-7 MΩ) were filled with (mmol/L) 122 K-aspartate, 8 KCl, 10 NaCl, 1 MgCl2, 10 HEPES, 5 Mg-ATP, (pH 7.2, KOH). The bath solution contained (mmol/L) 140 NaCl, 4 KCl, 1 MgCl2, 2 CaCl2, 10 glucose, 5 HEPES (pH 7.4, NaOH). Access resistance was typically <15 MΩ after patch rupture. Liquid junction potentials were corrected with the pipette in the bath. Fast capacitance was compensated in cell-attached configuration. Membrane capacitance and series resistance were compensated after patch rupture. Action potentials were continuously elicited in current clamp configuration by square current pulses of 1-2 nA amplitude and 1-5 ms duration at variable basic cycle lengths. Signals were filtered with a 2.9 kHz Bessel filter and recorded with an EPC10 amplifier (HEKA Elektronik). All experiments were conducted at room temperature.

Measurements of contractility in human atrial trabeculae
Trabeculae were dissected from right-atrial appendage biopsies. For isometric force recordings, trabeculae were mounted in a chamber and connected to a force transducer. Trabeculae were superfused with Krebs-Henseleit solution (in mmol/L):

NaCl 116, KCL 5, NaH_2PO_4 2, $MgCl_2$ 1.2, Na_2SO_4 1.2, $NaHCO_3$ 20, glucose 10) that was oxygenated with 95% O_2 and 5% CO_2 (37°C), and were electrically-field stimulated (1 Hz, pulse amplitude 60 mA, pulse width 50 ms). Before measurements, extracellular Ca^{2+} concentration was increased by 0.25 mmol/L steps every 2 min until the final concentration of 2 mmol/L was reached. After an equilibration period of 30 min, the trabeculae were gradually stretched until the maximum steady-state twitch force was achieved. After vehicle measurements, each trabecula was exposed to increasing concentrations of SAR296968, serving as its own vehicle control. SAR296968 was washed in for 20 min under ongoing electrical stimulation before steady-state was analyzed. Post-rest behavior was assessed by measuring the force after a 30s pause of electrical stimulation, whereas the ratio of the first contraction after the pause and the steady state before the pause was assessed. Developed force was normalized to the cross-sectional area of each trabecula (thickness x width x π/4) and expressed in mN/mm^2. For the analysis of the fractional difference in developed tension upon SAR296968, for each patient, we divided the mean developed tension upon SAR296968 by the mean developed tension upon corresponding vehicle and subtracted 1, then multiplied by 100 to obtain %. This results in a positive value if the developed tension was increased by SAR296968 in the specific patient, and a negative value if it was decreased. Afterwards, statistical analysis was conducted to test if the fractional difference data was significantly different from zero.

Supplemental Figure Legends:

Supplemental Figure S1. Additional Ca^{2+} spark parameters

Mean data per patient of Ca^{2+} spark full width half maximum (FWHM) (A), amplitude (B), full duration half maximum (FDHM) (C), and calculated SR Ca leak (D). Averaged caffeine-induced transient traces for the patients from analysis in figure 1C, main manuscript, shown in (E). p values are denoted above the corresponding groups, RM-mixed effects analysis with Bonferroni post-hoc test.

Supplemental Figure S2. Additional atrial trabeculae characteristics

Mean data per patient: A) Raw developed tension indicated in mN/mm^2 (p for linear trend p=0.296) B) The 80% return time to baseline tension (RT80) in (ms) (p for linear trend p=0.205).

Supplemental Figure S3. Additional AP characteristics

Mean data per patient: A) AP upstroke velocity and B) action potential duration (APD) at 50% of maximum repolarization were unaltered by SAR296968. p values are denoted above the corresponding groups, RM-mixed effects analysis with Bonferroni post-hoc test.

Supplemental Figure S4. Subgroup analyses of HFpEF patients

Mean data per patient: A) CaSpF was significantly decreased by SAR296968 in human atrial myocytes of HFpEF and non-HFpEF patients. B) Caffeine transient amplitude was significantly increased by SAR296968 in human atrial myocytes of non-HFpEF patients, a certain trend was visible for HFpEF. C) SAR296968 significantly reduced diastolic tension of human atrial trabeculae in HFpEF patients, a certain trend was also visible for non-HFpEF patients without statistical significance. p-values are denoted above the corresponding groups, RM-mixed effects analysis with Bonferroni post-hoc test.

References in the Supplemental Material

1. Cheng, H.; Lederer, W. J.; Cannell, M. B., Calcium sparks: elementary events underlying excitation-contraction coupling in heart muscle. *Science* **1993,** 262, (5134), 740-4. 10.1126/science.8235594
