# Supplementary figures and images for: SAR296968, a Novel Selective Na+/Ca2+ Exchanger Inhibitor, Improves Ca2+ Handling and Contractile Function in Human Atrial Cardiomyocytes
Source: Biomedicines. 2022 Aug 9;10(8):1932. doi: 10.3390/biomedicines10081932 (PMC9406204; doi:10.3390/biomedicines10081932)

# Supplemental Figure S1

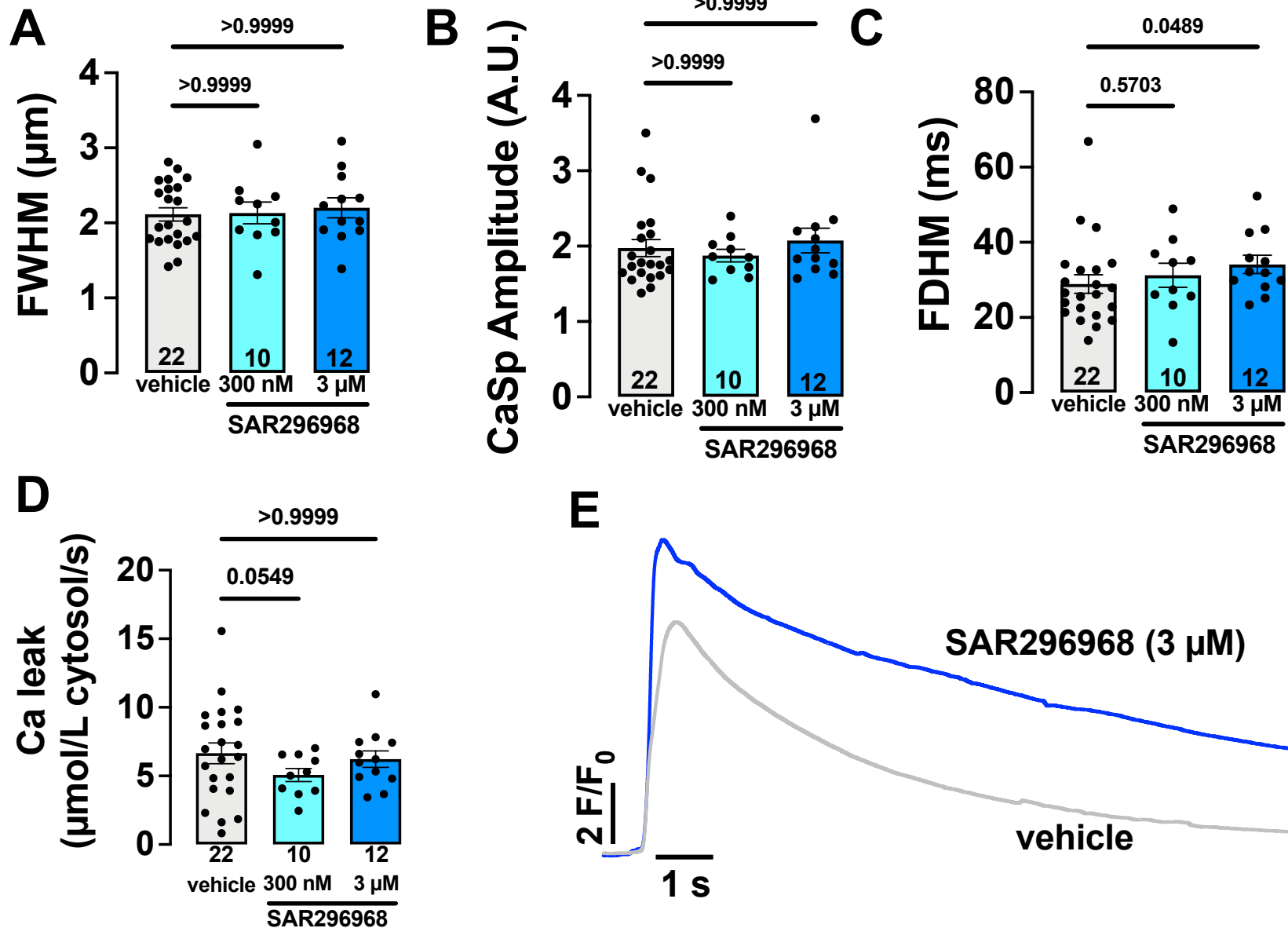

## Supplemental Figure S2

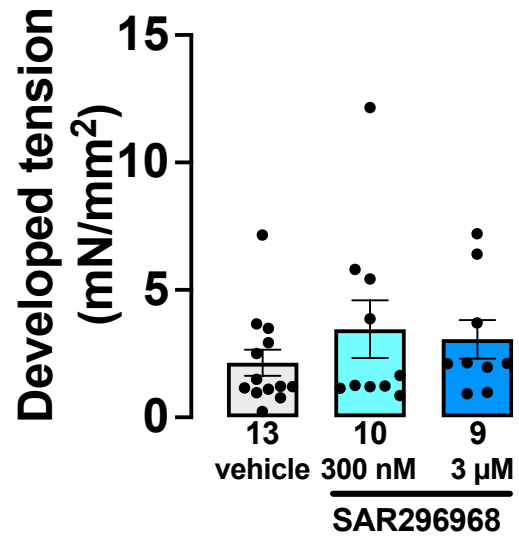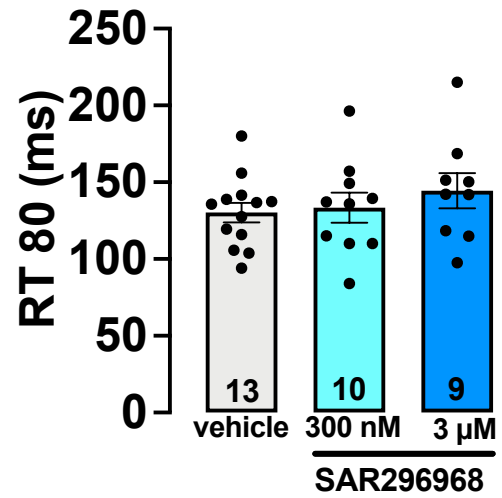

# Supplemental Figure S3

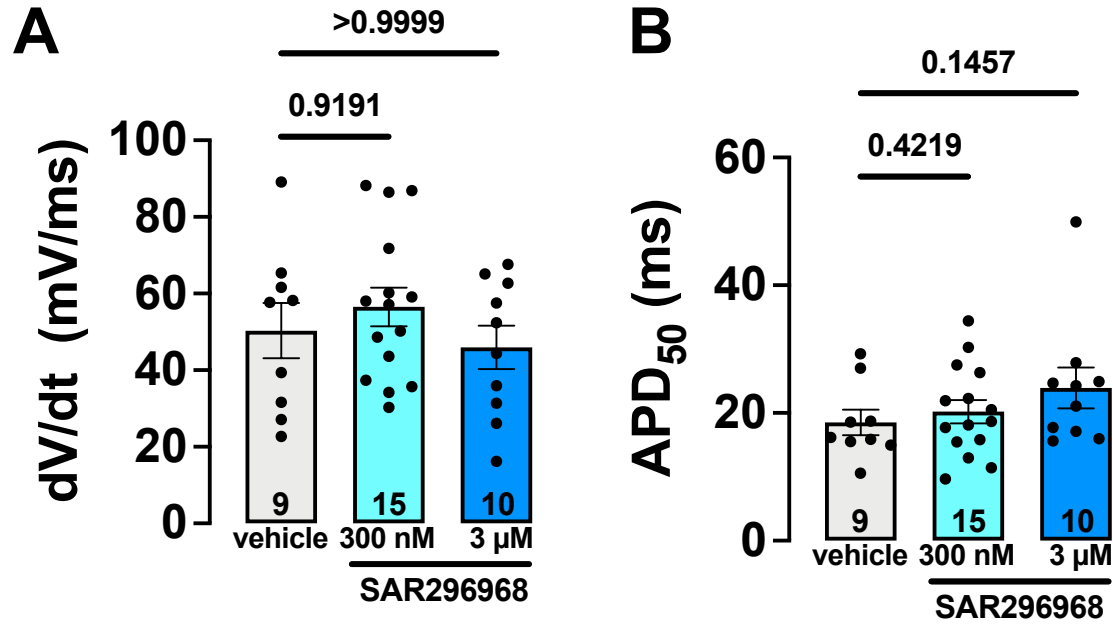

# Supplemental Figure S4

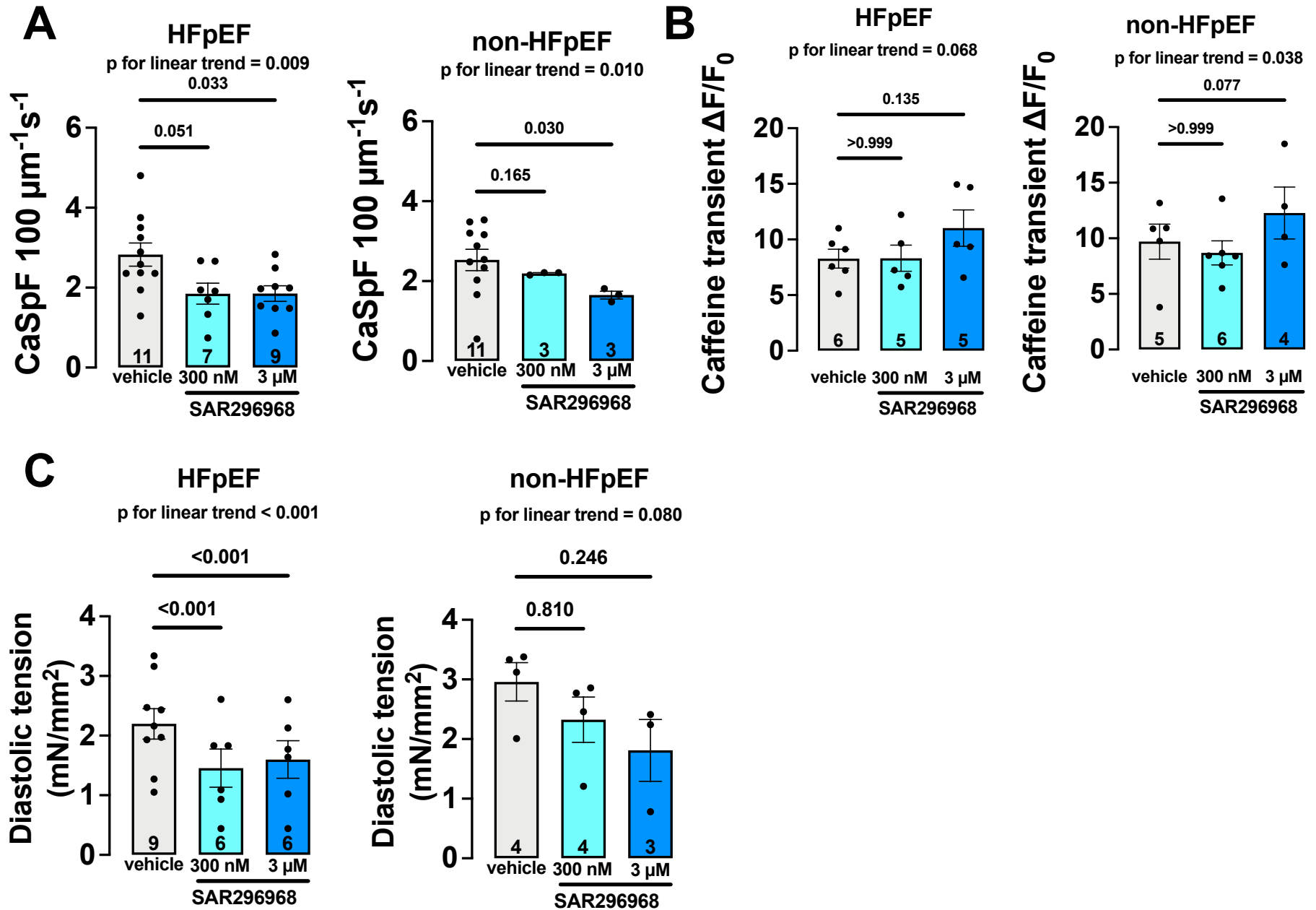

Supplement: Supplementary file 1 [file biomedicines-10-01932-s001.zip › Supplemental Figures_R1.pdf]
